# TRIM28-mediated p53 ubiquitination inhibits trophoblast apoptosis to support normal placental development

**DOI:** 10.1515/biol-2025-1236

**Published:** 2026-01-13

**Authors:** Huixiang Qian, Ruping Quan, Juan Chen, Yanghong Liu, Jing Hu, Ruizhen Li, Chao Lv

**Affiliations:** Department of Obstetrics and Gynecology, The Third Xiangya Hospital of Central South University, Changsha, 410013, Hunan Province, China; Institute of Reproductive & Stem Cell Engineering, Xiangya School of Basic Medical Sciences, Central South University, Changsha, 410013, Hunan Province, China; Outpatient Department, The Third Xiangya Hospital of Central South University, Changsha, 410013, Hunan Province, China

**Keywords:** TRIM28, pregnancy, placental development, p53 ubiquitination, apoptosis

## Abstract

Tripartite motif-containing 28 (TRIM28), a transcriptional regulatory factor, is involved in various biological processes. However, its role in early-onset preeclampsia (EOPE) remains unclear. An EOPE mouse model was established using Nω-Nitro-L-arginine methyl ester (L-NAME), while TRIM28 or tumor protein p53 (p53) expression was modulated through lentiviral infections. Maternal blood pressure and urinary protein levels were measured. Placental tissues were collected at embryonic day 17.5 for hematoxylin-eosin staining, immunohistochemistry, Western blot, and real-time quantitative polymerase chain reaction. *In vitro*, HTR-8/SVneo trophoblast cells were exposed to hydrogen peroxide (H_2_O_2_) to simulate oxidative stress. TRIM28 and p53 were overexpressed via lentiviral vectors, and cell apoptosis and molecular changes were assessed. TRIM28 expression was significantly downregulated in placentas from EOPE mice. Mechanistically, TRIM28 suppressed p53 level, downregulating pro-apoptotic proteins Bcl-2-associated *X* protein (Bax) and cleaved caspase-3, while upregulating the anti-apoptotic protein B-cell lymphoma-2 (Bcl-2). In trophoblast cells, TRIM28 alleviated H_2_O_2_-induced apoptosis by promoting p53 ubiquitination and thereby reducing its pro-apoptotic activity. TRIM28 attenuates oxidative stress-induced trophoblast apoptosis and may help protect against the development of EOPE.

## 1 Introduction

Early-onset preeclampsia (EOPE) is a serious pregnancy complication that typically arises before 34 weeks of gestation [1]. It is characterized by hypertension and proteinuria and is associated with significant maternal and fetal morbidity and mortality [2]. EOPE is associated with poor placental development, which plays a key role in its pathogenesis [3]. Proper placental development relies on adequate trophoblast invasion and differentiation [4]. In EOPE, these processes are frequently disrupted, leading to inadequate remodeling of uterine spiral arteries and poor placental perfusion [5]. Excessive trophoblast apoptosis is a common pathological feature in EOPE placentas, causing structural damage and functional impairment [6]. However, the molecular mechanisms that regulate trophoblast apoptosis in EOPE remain incompletely understood, and further research is needed to identify key regulatory factors and signaling pathways.

Tripartite motif-containing 28 (TRIM28) is a transcriptional corepressor widely expressed in various tissues and known to regulate gene expression by interacting with transcription factors and epigenetic regulators [7]. TRIM28 interacts with tumor protein p53 (p53), a critical tumor suppressor, and modulates its stability and activity through post-translational modifications such as ubiquitination [8]. However, whether this mechanism occurs in EOPE and how it affects trophoblast survival remains unknown. Under normal physiological conditions, p53 maintains cellular homeostasis by responding to stress signals and controlling pro- and anti-apoptotic genes [9]. In pathological conditions such as EOPE, dysregulated p53 expression and activity may contribute to excessive trophoblast apoptosis [10].

Based on these findings, we hypothesize that TRIM28 may protect against excessive trophoblast apoptosis in EOPE by promoting p53 ubiquitination and reducing its pro-apoptotic activity. This study aims to explore the role of TRIM28 in regulating trophoblast apoptosis via the p53 signaling pathway in EOPE. Understanding this mechanism may provide new insights into the molecular basis of EOPE and offer potential therapeutic targets to improve pregnancy outcomes.

## 2 Materials and methods

### 2.1 Animal modeling and grouping

Female C57BL/6 mice were housed under standard laboratory conditions with a 12-h light/dark cycle, temperature maintained at 18–22 °C, and relative humidity of 50 %. Animals had *ad libitum* access to food and water. Mice were divided into six groups (*n* = 6 per group): Normal control, Nω-Nitro-L-arginine methyl ester (L-NAME), lentiviral vector overexpressing negative control (oe-NC), lentiviral vector overexpressing TRIM28 (oe-TRIM28), oe-TRIM28 + oe-NC, and oe-TRIM28 + lentiviral vector overexpressing p53 (oe-p53). To acclimate mice to blood pressure monitoring, a seven-stage training protocol was performed with the CODA non-invasive tail-cuff system (Kent Scientific, Torrington, CT, USA), progressively familiarizing them with the restraining tube and tail cuff. For *in vivo* gene manipulation, lentiviral vectors with oe-NC, oe-TRIM28, or oe-p53 were injected into the uterine lumen on embryonic day 3.0 (E3.0) [11]. From E7.5 through E17.5, EOPE was induced by daily subcutaneous injections of L-NAME (50 mg/kg/day in 100 μL phosphate buffer saline [PBS]) or PBS alone (as control) [12]. Urine samples were collected and maternal blood pressure was measured on E17.5. On embryonic day 17.5, mice were sacrificed by intraperitoneal administration of sodium pentobarbital (150 mg/kg), and placental tissues were collected for subsequent analysis.

**Ethical approval:** The research related to animal use has been complied with all the relevant national regulations and institutional policies for the care and use of animals, and has been approved by the Animal Committee of the Third Xiangya Hospital of Central South University (No. 2023-S117).

### 2.2 Cell culture

The human extravillous trophoblast cell line HTR-8/SVneo (American Type Culture Collection [ATCC], Manassas, VA, USA) was cultured in Roswell Park Memorial Institute (RPMI) 1,640 medium (Gibco, Thermo Fisher Scientific Inc., Waltham, MA, USA) at 37 °C in 5 % CO_2_). To mimic oxidative stress, cells were exposed to hydrogen peroxide (H_2_O_2_) at a concentration of 0.1 mM for 24 h.

### 2.3 Lentiviral transduction

Lentiviruses encoding TRIM28, p53, or NC sequences were used for gene overexpression. HTR-8/SVneo cells were seeded at a density of 1 × 10^5^ cells per well in 24-well plates. At approximately 60 % confluency, lentiviral particles were added to the culture medium. Cells were harvested 48 h after infection for downstream experiments.

### 2.4 Enzyme-linked immunosorbent assay (ELISA)

Urine samples were centrifuged at 800×*g* for 5 min to remove cellular debris. Albumin levels were measured with a mouse albumin ELISA kit (MU30662, Bioswamp, Wuhan, China) according to the manufacturer’s instructions. Absorbance was recorded at 450 nm using a microplate reader, and albumin concentrations were then calculated.

### 2.5 Hematoxylin and eosin (HE) staining

Placental tissues were fixed, paraffin-embedded, and sectioned at 4 µm thickness. Sections were dewaxed in xylene (Sigma-Aldrich, St. Louis, MO, USA), rehydrated through graded ethanol, and rinsed in distilled water. Hematoxylin staining was followed by eosin counterstaining. After dehydration in ascending ethanol concentrations and xylene clearing, sections were mounted in neutral resin. Morphological evaluation was performed with an optical microscope (CX43, Olympus Corporation, Tokyo, Japan).

### 2.6 Immunohistochemistry (IHC)

After deparaffinization, endogenous peroxidase activity was blocked using 3 % H_2_O_2_ for 10 min. Antigen retrieval was performed by microwave heating in citrate buffer (two cycles, 3 min each). Sections were blocked and incubated overnight at 4 °C with the following primary antibodies targeting cleaved caspase-3 (1:400, 9,661, Cell Signaling Technology, Shanghai, China), Bcl-2-associated *X* protein (Bax, 1:100, A12009, Abclonal, Wuhan, China), B-cell lymphoma 2 (Bcl-2, 1:1,000, A19693, Abclonal), TRIM28 (1:200, A19568, Abclonal), and p53 (1:1,000, A25915, Abclonal). Sections were incubated for 1 h with horseradish peroxidase (HRP)-conjugated goat anti-rabbit secondary antibody (1:2000, ab205718, Abcam, Cambridge, UK). Visualization was achieved using 3,3′-diaminobenzidine (DAB) substrate solution (P0203, Beyotime, Shanghai, China), followed by hematoxylin counterstaining. The stained tissue samples were observed with an optical microscope (CX43, Olympus, Tokyo, Japan), and the areas showing positive staining were measured as a percentage of the total tissue area using ImageJ software (National Institutes of Health, Bethesda, MD, USA).

### 2.7 Measurement of reactive oxygen species (ROS)

Tissue ROS levels were measured using a detection kit (KL1470516A, KANLANG, China). After washing, sections were incubated with diluted BBoxiProbe™ DHE working solution at 37 °C in the dark for 60 min. The sections were then washed with PBS, mounted, and observed under a fluorescence microscope to detect ROS signals. Cellular ROS levels were measured using a detection kit (MB4682, Meilunbio, Dalian, China). A 5 μM 2′,7′-dichlorodihydrofluorescein diacetate (DCFH-DA) working solution was freshly prepared prior to the experiment. Suspension or adherent cells were incubated with the working solution for 30 min, washed with PBS, and analyzed under a fluorescence microscope.

### 2.8 Real-time quantitative polymerase chain reaction (RT-qPCR)

RNA was extracted from both tissue samples and cultured cells utilizing a rapid RNA isolation kit (DP419, Jiachu Biotechnology Co., Ltd., Shanghai, China). Complementary DNA (cDNA) was synthesized and subjected to RT-qPCR using a fluorescence-based quantification kit (QR0100, Sigma-Aldrich). Glyceraldehyde-3-phosphate dehydrogenase (GAPDH) served as the internal reference. Relative mRNA levels were calculated using the 2^–ΔΔCt^ method. The primer sequences used are listed in Table 1.

**Table 1:** Primer sequences.

Gene	Species	Direction	Sequence (5′-3′)
TRIM28	*Mus musculus*	Forward	CTG​CTG​CCC​TGT​CTA​CAT​TCG
		Reverse	ACA​CTG​GAC​AAT​CCA​CCA​TAG​C
	*Homo sapiens*	Forward	GTC​AAT​GAT​GCC​CAG​AAG​GTG​A
		Reverse	CAA​AAG​GGC​TGT​GTT​GTT​GTC​A
p53	*Mus musculus*	Forward	TTC​TCC​GAA​GAC​TGG​ATG​ACT​G
		Reverse	AAA​ATG​TCT​CCT​GGC​TCA​GAG​G
	*Homo sapiens*	Forward	CTC​AAA​AGT​CTA​GAG​CCA​CCG​T
		Reverse	CAG​TCT​GGC​TGC​CAA​TCC​A
GAPDH	*Mus musculus*	Forward	GCC​TCC​TCC​AAT​TCA​ACC​CTT​A
		Reverse	TTT​GTC​TAC​GGG​ACG​AGG​AAA​C
	*Homo sapiens*	Forward	GTC​TCC​TCT​GAC​TTC​AAC​AGC​G
		Reverse	ACC​ACC​CTG​TTG​CTG​TAG​CCA​A

### 2.9 Western Blot (WB)

Total cellular proteins were extracted using radioimmunoprecipitation assay (RIPA) lysis buffer (ab170197, Abcam). Protein levels were measured using the bicinchoninic acid (BCA) assay kit (P0010, Beyotime, Shanghai, China). Equivalent protein quantities were resolved by Sodium Dodecyl Sulfate Polyacrylamide Gel Electrophoresis and then transferred onto polyvinylidene fluoride (PVDF) membranes (ab133411, Abcam) using a wet-transfer apparatus. The membranes were blocked with 5 % non-fat milk and incubated overnight at 4 °C with the designated primary antibodies: anti-TRIM28 (1:1,000, A19568, Abclonal, Wuhan, China), anti-cleaved caspase-3 (1:1,000, 9,661, Cell Signaling Technology, Shanghai, China), anti-Bax (1:1,000, A12009, Abclonal), anti-Bcl-2 (1:1,000, A19693, Abclonal), anti-p53 (1:1,000, A25915, Abclonal), and anti-GAPDH (glyceraldehyde-3-phosphate dehydrogenase, 1:2000, ab181602, Abcam). Following incubation with HRP-conjugated goat anti-rabbit secondary antibody, protein bands were visualized and quantified using ImageJ software.

### 2.10 Cell viability

HTR-8/SVneo cells were seeded in 96-well plates at a density of 5,000 cells per well. A 10 % solution of Cell Counting Kit-8 (CCK-8) reagent (HY–K0301, MedChemExpress, Monmouth Junction, NJ, USA) was added to each well and incubated at 37 °C for 3 h. Subsequently, cell viability was assessed by measuring the absorbance at 450 nm with a microplate reader (Thermo Fisher Scientific).

### 2.11 Terminal deoxynucleotidyl transferase dUTP nick end labeling (TUNEL) assay

TUNEL apoptosis detection kit (C1089, Beyotime) was applied. Cells were fixed in 4 % paraformaldehyde for 30 min and permeabilized with a potent immunostaining permeabilization solution (P0097, Beyotime) for 5 min at room temperature. Subsequently, cells were treated with 50 µL of TUNEL reaction solution and incubated in the dark at 37 °C for 1 h. Afterward, nuclei were stained using 4′,6-diamidino-2-phenylindole (DAPI). Slides were mounted using anti-fade mounting medium and observed under a fluorescence microscope. Apoptosis was quantified as the percentage of TUNEL-positive cells using ImageJ software.

### 2.12 Co-immunoprecipitation (Co-IP)

Cells were lysed using Pierce IP lysis buffer (87787, Thermo Fisher Scientific) with a protease inhibitor cocktail (Roche, Basel, Switzerland) to extract total proteins. The lysates were incubated overnight at 4 °C with anti-TRIM28 antibody (3 µg, A2245, Abclonal) or control rabbit immunoglobulin G (1 µg, 30000-0-AP, Proteintech, Wuhan, China) pre-coupled to Protein A/G PLUS-Agarose beads (20423, Thermo Fisher Scientific). The immune complexes were collected, boiled in sample loading buffer, and analyzed by WB.

### 2.13 Ubiquitination assay

Cells were transfected individually or co-transfected with Myc-tagged p53, hemagglutinin (HA)-tagged ubiquitin (Ub), and Flag-tagged TRIM28 plasmids. After transfection, cells were lysed using RIPA buffer containing protease inhibitors. The supernatant was collected following centrifugation to remove cell debris and subjected to immunoprecipitation with anti-HA antibody. WB assessed the levels of p53 ubiquitination in the presence or absence of TRIM28 by detecting the expression of HA (1:1,000, ab236632, Abcam), Myc (1:250, ab9106, Abcam), and Flag (1:1,000, SAB4301135, Thermo Fisher Scientific) antibodies.

### 2.14 Protein stability assay

To evaluate protein degradation dynamics, cells transfected with oe-TRIM28 were treated with cycloheximide (CHX; 20 μg/mL, Sigma-Aldrich) to block new protein synthesis. Cells were harvested at 0, 15, 30, 60, 120, and 240 min post-treatment, lysed, and subjected to WB to assess changes in protein stability over time.

### 2.15 Statistics

Statistical analyses were conducted using GraphPad Prism 9 (Dotmatics, Boston, MA, USA). Data normality was assessed before analysis. For comparisons involving multiple groups, one-way or two-way analysis of variance (ANOVA) with Tukey’s post hoc test was used. Differences between two groups were evaluated using Student’s *t*-test. A *p*-value < 0.05 was considered statistically significant. Each experiment was independently replicated at least three times to ensure reproducibility.

## 3 Results

### 3.1 TRIM28 is downregulated in placental tissues following L-NAME treatment

**Figure 1:**
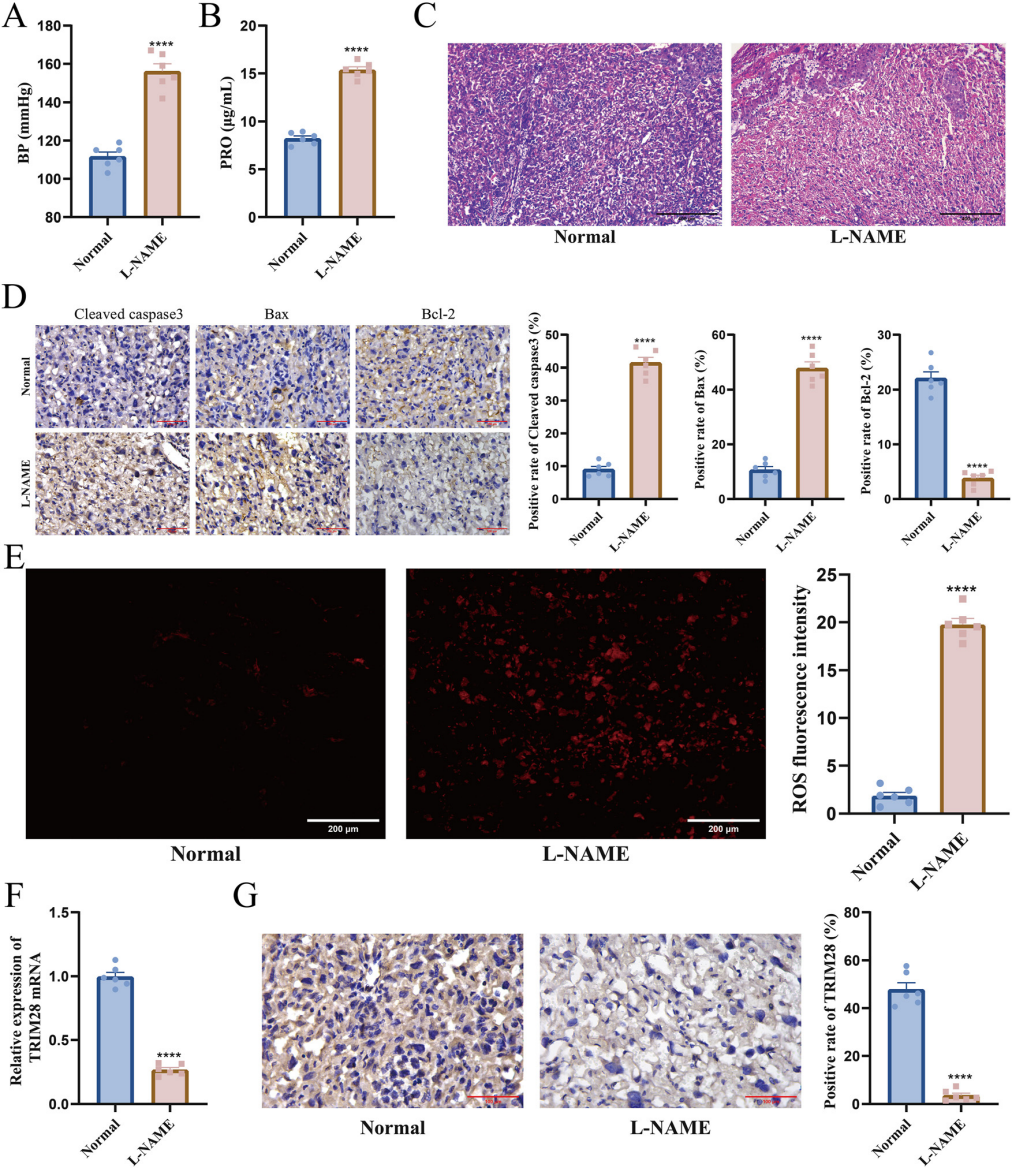
Expression of TRIM28 and related indicators in placentas of L-NAME-induced EOPE mice. (A) Measurement of systolic blood pressure in mice using the tail-cuff method; (B) determination of urinary protein levels using the BCA assay; (C) HE staining observation of placental tissue structure, the scale bar represents 400 μm; (D) IHC detection of cleaved caspase-3, Bax, and Bcl-2 protein expression, the scale bar represents 100 μm; (E) measurement of ROS levels in placental tissue, the scale bar represents 200 μm; (F) analysis of TRIM28 mRNA expression in placenta by RT-qPCR; (G) IHC evaluation of TRIM28 protein expression in placental tissue, the scale bar represents 100 μm. *N* = 6. ****p < 0.0001.

To investigate TRIM28 expression in abnormal placental development, an EOPE mouse model was established using L-NAME. L-NAME significantly increased systolic blood pressure (Figure 1A) and urinary protein levels (Figure 1B), confirming successful model establishment. HE staining showed disorganized placental villi, irregular trophoblast arrangement, and focal necrosis in the L-NAME-treated mice (Figure 1C). L-NAME treatment also increased expression of pro-apoptotic proteins cleaved caspase-3 and Bax, along with decreased Bcl-2 level (Figure 1D) and elevated ROS levels (Figure 1E). RT-qPCR exhibited decrease of TRIM28 mRNA (Figure 1F), while IHC confirmed reduced TRIM28 protein levels (Figure 1G). These findings indicate that TRIM28 downregulation is associated with oxidative stress and increased apoptosis in the placenta of L-NAME-induced EOPE mice.

### 3.2 TRIM28 overexpression alleviates L-NAME-induced placental injury

**Figure 2:**
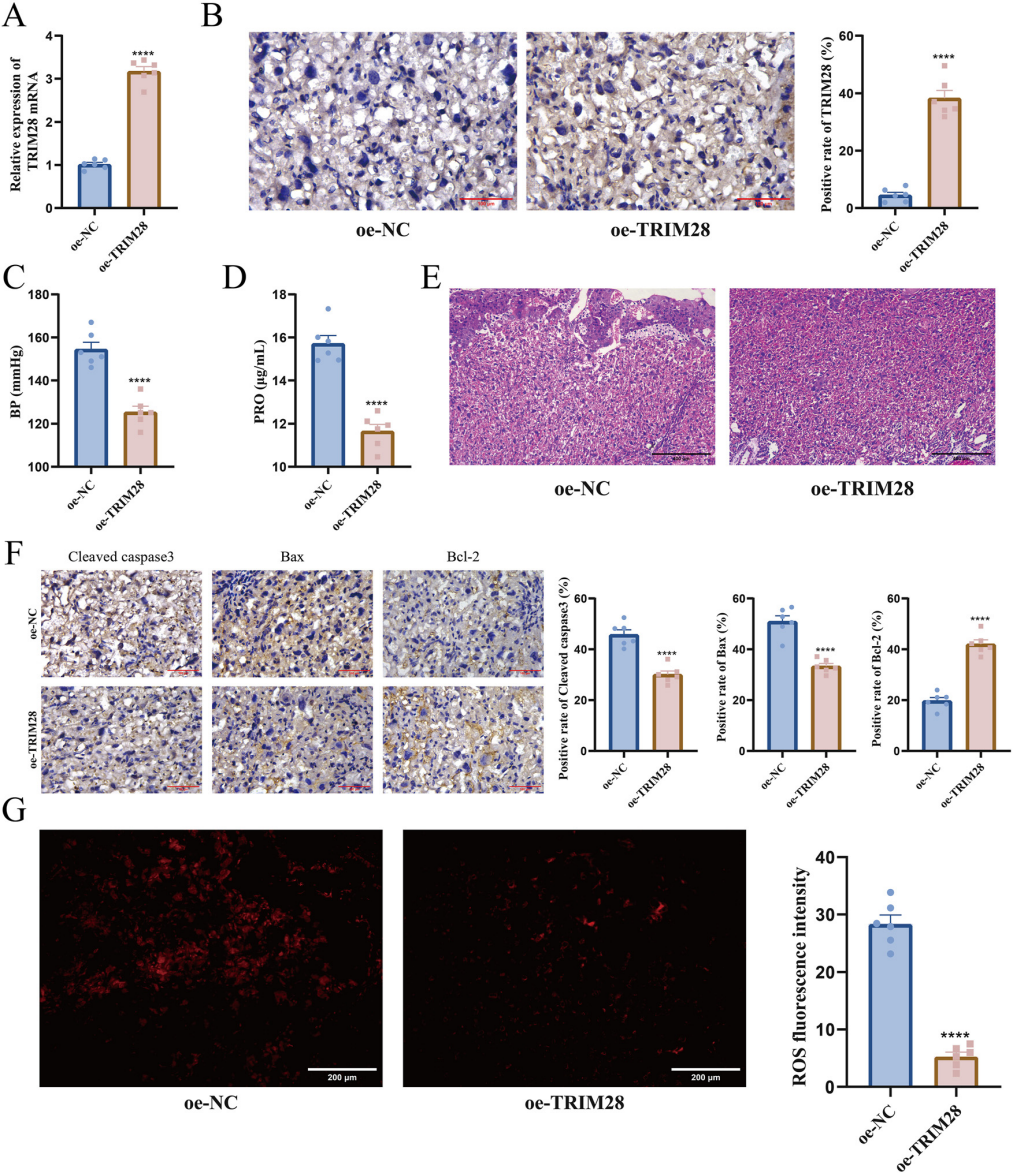
Alleviation of L-NAME-induced placental injury by TRIM28 overexpression. (A) RT-qPCR detection of TRIM28 mRNA expression in placental tissue; (B) IHC detection of TRIM28 protein expression in placenta, the scale bar represents 100 μm; (C) measurement of systolic blood pressure in pregnant mice; (D) urinary protein detection to evaluate renal impairment; (E) HE staining for histological observation of placental structure, the scale bar represents 400 μm; (F) IHC detection of cleaved caspase-3, Bax, and Bcl-2 expression to assess apoptosis, the scale bar represents 100 μm; (G) measurement of ROS levels in placental tissue to evaluate oxidative stress, the scale bar represents 200 μm. *N* = 6. ****p < 0.0001.

To determine whether TRIM28 upregulation could protect against placental injury, EOPE mice were treated with oe-TRIM28 vectors. RT-qPCR and IHC confirmed significantly increased TRIM28 expression in the placenta of the oe-TRIM28-treated EOPE mice (Figures 2A and B). TRIM28 overexpression reduced systolic blood pressure and urinary protein levels (Figure 2C and D), indicating improved renal function. Placental structure was preserved, with intact villi and normal trophoblast morphology (Figure 2E). In addition, cleaved caspase-3 and Bax levels were reduced, while Bcl-2 was restored (Figure 2F), suggesting suppression of apoptosis. Moreover, ROS levels were significantly reduced following TRIM28 overexpression (Figure 2G).

### 3.3 TRIM28 overexpression attenuates H_2_O_2_-induced apoptosis in HTR-8/SVneo cells

**Figure 3:**
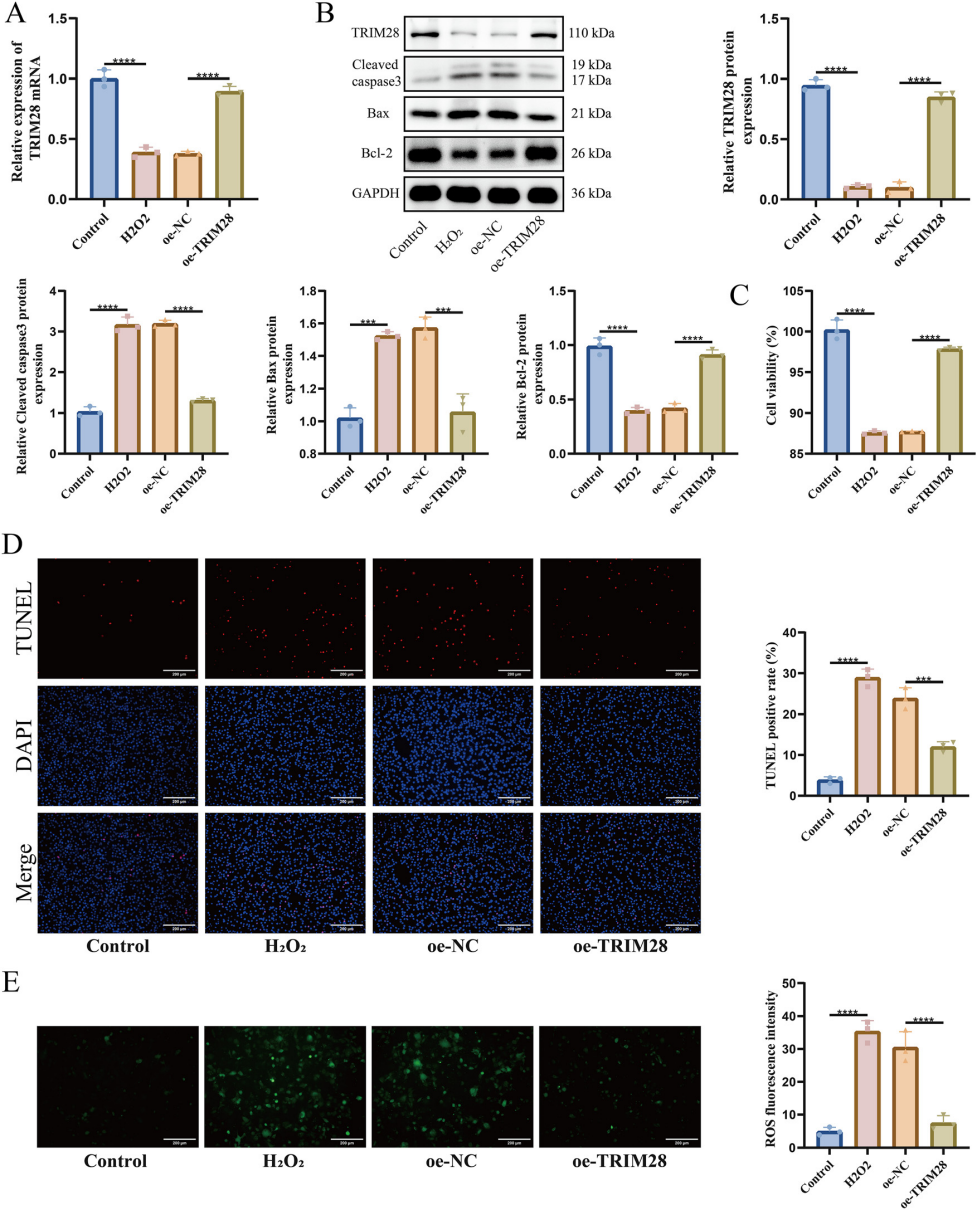
Reduction of H_2_O_2_-induced apoptosis in HTR-8/SVneo cells by TRIM28 overexpression. (A) RT-qPCR detection of TRIM28 mRNA expression; (B) WB detection of TRIM28, cleaved caspase-3, Bax, and Bcl-2 protein expression; (C) CCK-8 assay for evaluation of cell viability; (D) TUNEL staining for detection of apoptotic cells, the scale bar represents 200 μm; (E) ROS level detection, the scale bar represents 200 μm. *N* = 3. ***p < 0.001, ****p < 0.0001.

To further verify TRIM28’s role in apoptosis, H_2_O_2_-induced injury was modeled in HTR-8/SVneo human trophoblast cells. H_2_O_2_ treatment downregulated TRIM28 mRNA, while TRIM28 overexpression reversed this effect (Figure 3A), which was confirmed at the protein level by WB (Figure 3B). H_2_O_2_ suppressed cell viability, whereas TRIM28 overexpression partially restored cell viability (Figure 3C). TUNEL staining indicated a marked increase in apoptosis after H_2_O_2_ exposure, which was reduced by TRIM28 overexpression (Figure 3D). WB analysis showed that H_2_O_2_ elevated cleaved caspase-3 and Bax while reducing Bcl-2 expression, but these changes were reversed in oe-TRIM28-infected cells (Figure 3B). TRIM28 overexpression also alleviated oxidative stress induced by H_2_O_2_ (Figure 3E).

### 3.4 p53 is upregulated in both L-NAME-treated placenta and H_2_O_2_-induced HTR-8/SVneo cells

**Figure 4:**
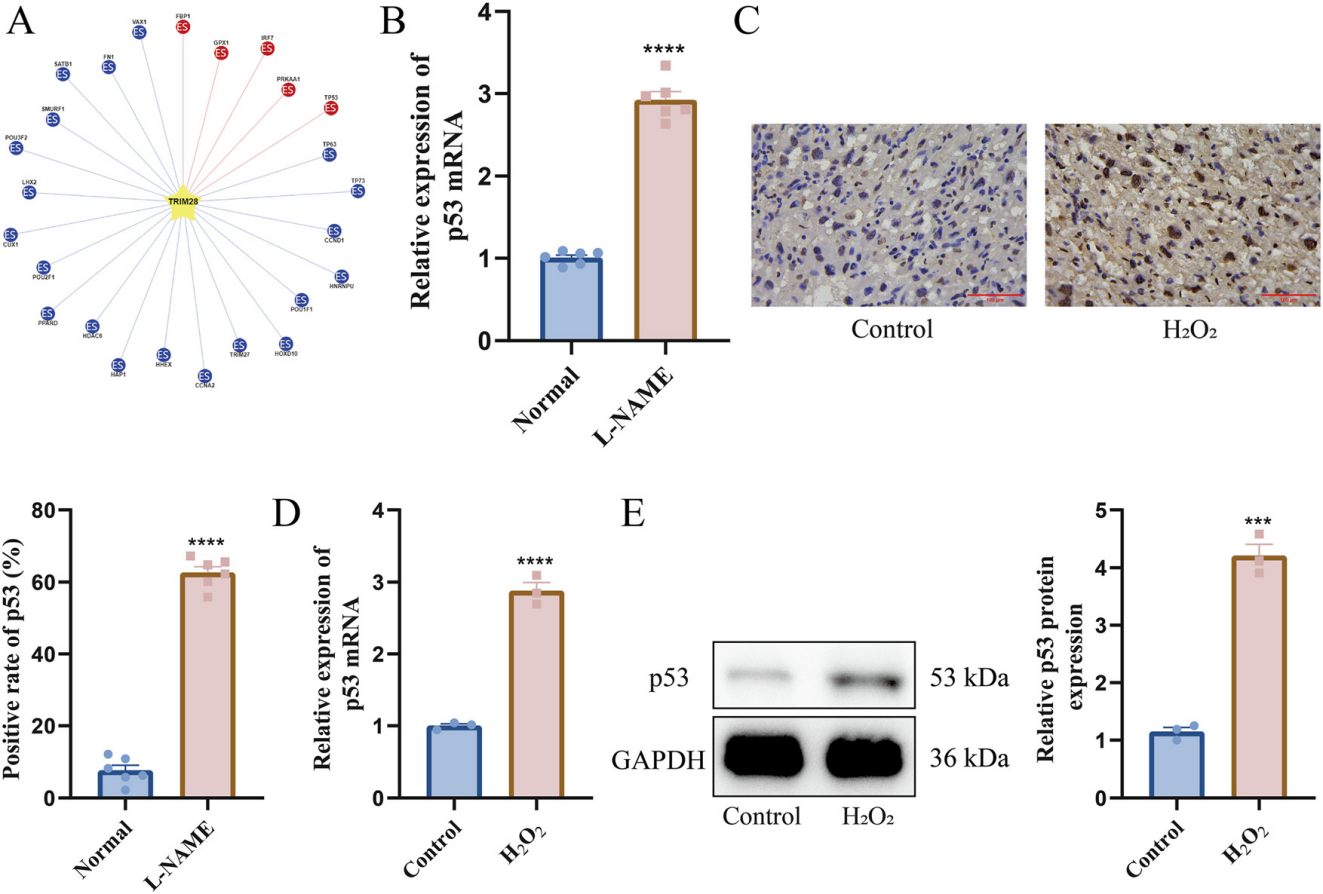
Upregulation of p53 in placentas of L-NAME-treated mice and H_2_O_2_-stimulated HTR-8/SVneo cells. (A) Prediction of TRIM28 downstream targets using the UbiBrowser database; (B) RT-qPCR detection of p53 mRNA expression in mouse placental tissue; (C) IHC detection of p53 protein expression in mouse placental tissue, the scale bar represents 100 μm; (D) RT-qPCR detection of p53 mRNA expression in H_2_O_2_-treated HTR-8/SVneo cells; (E) WB detection of p53 protein expression in H_2_O_2_-treated HTR-8/SVneo cells. *N* = 6 (B–C), *N* = 3 (D–E). ***p < 0.001, ****p < 0.0001.

UbiBrowser database (http://ubibrowser.bio-it.cn/ubibrowser_v3/home/index) identified p53 as a downstream target of TRIM28, known to regulate Bax and Bcl-2 (Figure 4A). RT-qPCR showed significant upregulation of p53 mRNA in L-NAME-treated mouse placentas (Figure 4B), and IHC confirmed increased p53 protein expression (Figure 4C). Similarly, H_2_O_2_-treated HTR-8/SVneo cells showed elevated p53 mRNA and protein levels (Figure 4D and E), suggesting that p53 may mediate TRIM28-associated apoptosis regulation.

### 3.5 TRIM28 promotes p53 ubiquitination

**Figure 5:**
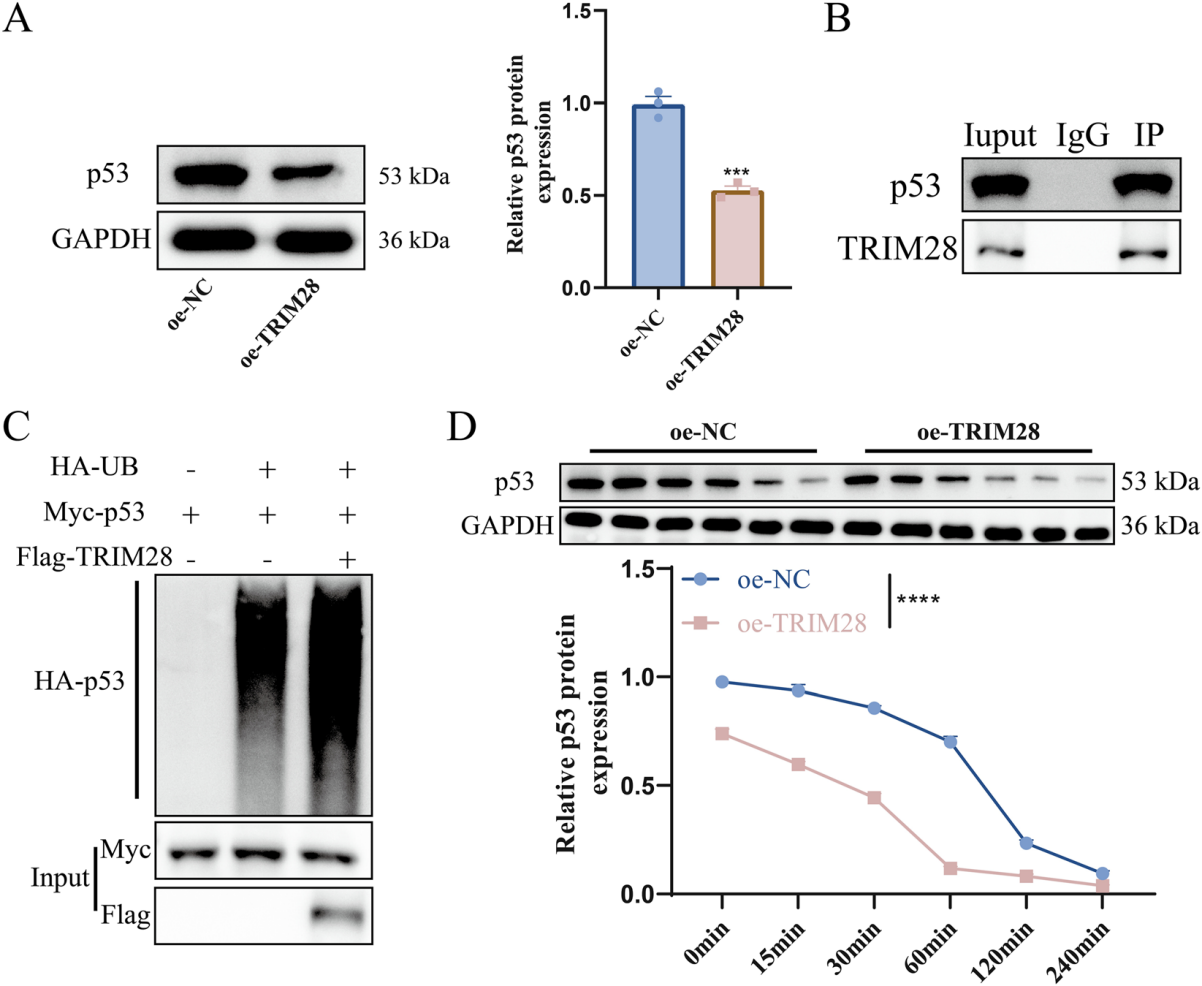
Ubiquitination regulation of p53 by TRIM28. (A) WB analysis of p53 protein expression in oe-TRIM28 and oe-NC groups; (B) Co-IP detection of TRIM28–p53 interaction; (C) Co-IP detection of p53–Ub binding and assessment of TRIM28’s effect on p53 ubiquitination; (D) evaluation of p53 protein stability following CHX treatment. *N* = 3. ***p < 0.001, ****p < 0.0001.

WB revealed reduced p53 protein levels following TRIM28 overexpression (Figure 5A). Co-IP confirmed the interaction between TRIM28 and p53 (Figure 5B). In HTR-8/SVneo cells co-transfected with HA-UB, Myc-p53, and Flag-TRIM28 plasmids and treated with MG132, TRIM28 markedly enhanced the binding between p53 and ubiquitin, indicating that TRIM28 promotes p53 ubiquitination (Figure 5C). CHX treatment further showed accelerated p53 degradation in oe-TRIM28 cells, confirming that TRIM28 reduced p53 stability via ubiquitination (Figure 5D).

### 3.6 TRIM28 alleviates excessive apoptosis and supports embryonic development by regulating p53 ubiquitination

**Figure 6:**
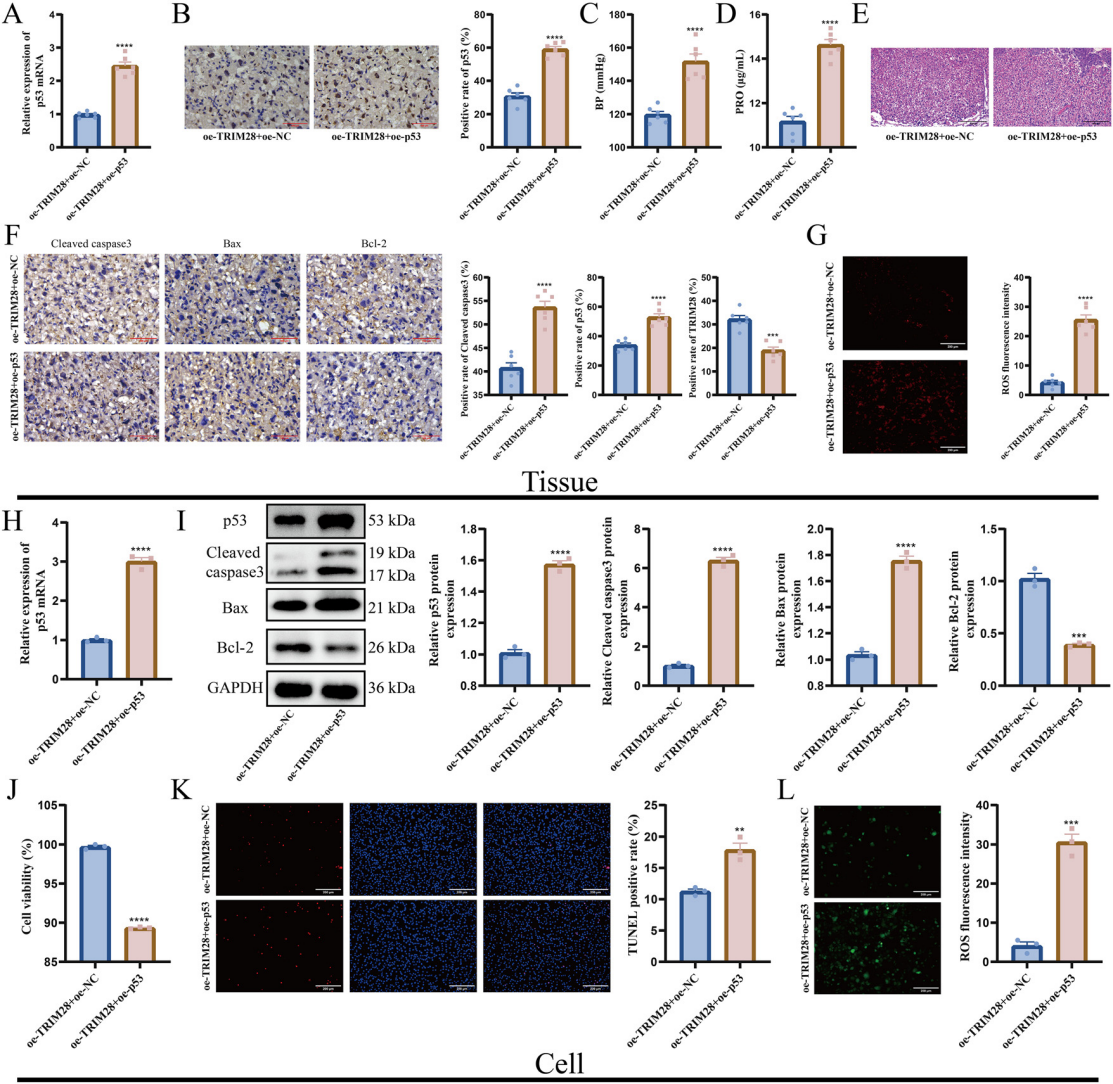
Effects of p53 overexpression on placental tissue and HTR-8/SVneo cells. (A) RT-qPCR detection of p53 mRNA expression in mouse placental tissue; (B) WB detection of p53 protein expression in mouse placental tissue, the scale bar represents 100 μm; (C) measurement of systolic blood pressure in mice; (D) detection of urinary protein levels; (E) HE staining for histological analysis of placental tissue, the scale bar represents 400 μm; (F) IHC detection of cleaved caspase-3, Bax, and Bcl-2 expression in placenta, the scale bar represents 100 μm; (G) detection of ROS levels in placental tissue; (H) RT-qPCR detection of p53 mRNA expression in HTR-8/SVneo cells; (I) WB detection of p53, cleaved caspase-3, Bax, and Bcl-2 protein expression in HTR-8/SVneo cells; (J) CCK-8 assay for assessing HTR-8/SVneo cell viability; (K) TUNEL staining for detection of apoptosis in HTR-8/SVneo cells, the scale bar represents 200 μm; (L) detection of ROS levels in HTR-8/SVneo cells, the scale bar represents 200 μm. *N* = 6 (A–G), *N* = 3 (H–L). **p < 0.01, ***p < 0.001, ****p < 0.0001.

In mouse placentas, p53 overexpression significantly increased both mRNA and protein levels of p53 (Figure 6A and B), elevated blood pressure (Figure 6C), and increased urinary protein levels (Figure 6D). HE staining showed disrupted placental architecture and impaired development (Figure 6E). Cleaved caspase-3 and Bax were upregulated, Bcl-2 was downregulated (Figure 6F). ROS levels were also elevated, indicating enhanced oxidative stress (Figure 6G). In HTR-8/SVneo cells, p53 overexpression increased expression of p53 (Figure 6H and I), reduced cell viability (Figure 6J), and elevated apoptosis (Figure 6K). WB further demonstrated elevated levels of cleaved caspase-3 and Bax, accompanied by a reduction in Bcl-2 expression (Figure 6I). ROS levels were also significantly increased by p53 overexpression (Figure 6L).

## 4 Discussion

The progression of EOPE remains challenging to predict, as conventional diagnostic markers such as hypertension and proteinuria often fail to accurately reflect disease severity or reliably forecast maternal and fetal outcomes [13]. This diagnostic uncertainty complicates clinical decision-making, particularly in determining the optimal timing of delivery. As such, there is an urgent need for novel molecular markers and therapeutic targets that can both clarify disease mechanisms and offer potential interventions. In this context, our research highlights TRIM28 as a critical regulator of trophoblast function and placental homeostasis.

Initially, our study demonstrated that TRIM28 expression was significantly downregulated in the placenta of EOPE mice induced by L-NAME, a commonly used method for inducing PE [14]. This mirrors previous observations in inflammatory disorders where reduced TRIM28 expression sensitized tissues to cellular stress [15], 16]. The parallel with TRIM72, another TRIM family protein shown to be reduced in PE [17], suggests that downregulation of TRIM proteins may represent a broader pathological signature in hypertensive disorders of pregnancy. Importantly, TRIM28 downregulation correlated with increased oxidative stress, apoptosis, and structural placental damage, strongly implicating its role in disease pathogenesis. Additionally, we observed elevated cleaved caspase-3 expression in EOPE placentas and H_2_O_2_-treated trophoblasts, which was reversed by TRIM28 overexpression. Cleaved caspase-3 is a key executioner caspase in apoptosis, and its increase reflects enhanced apoptotic activity [18]. Our results suggested that TRIM28 inhibited caspase-3 activation, possibly by suppressing upstream pro-apoptotic signaling. Similarly, the increase in Bax and reduction in Bcl-2 expression following L-NAME or H_2_O_2_ treatment indicate a shift toward a pro-apoptotic environment, as Bax exerts pro-apoptotic function while Bcl-2 opposes apoptosis [19]. TRIM28 overexpression restored the Bax/Bcl-2 balance, implying its regulatory role in apoptosis. Another key finding is the ability of TRIM28 to reduce ROS accumulation in both *in vivo* and *in vitro* models. This is consistent with findings that TRIM28 can mitigate oxidative stress by regulating antioxidant pathways. Elevated ROS is a hallmark of EOPE pathology, driving placental ischemia-reperfusion injury, DNA damage, and trophoblast apoptosis [20]. TRIM28’s antioxidative role may involve transcriptional regulation of antioxidant genes or suppression of p53-mediated ROS generation. Previous evidence suggests TRIM28 can coordinate chromatin remodeling and transcription factor recruitment [21]. The ability of TRIM28 to reduce ROS suggests a possible mechanism through which it preserves trophoblast viability and placental integrity.

Our mechanistic investigation revealed that TRIM28 directly regulates p53 stability by promoting its ubiquitination. Elevated p53 levels in EOPE placentas and H_2_O_2_-treated trophoblasts correlated with heightened apoptosis and ROS, consistent with the pro-apoptotic function of p53 in trophoblasts [22]. Besides, the p53 pathway’s abnormal protein expression drives excessive trophoblast apoptosis in PE, and modulating this pathway can alter the apoptotic process [23]. In our study, p53 expression was markedly elevated in placental tissues of L-NAME-induced EOPE mice and H_2_O_2_-treated HTR-8/SVneo cells, further suggesting a role in stress-induced apoptosis. These results agree with studies identifying p53 as a pro-apoptotic factor in trophoblasts by upregulating Bax and downregulating Bcl-2 [24]. Moreover, we detected that TRIM28 overexpression led to increased binding between p53 and ubiquitin, accelerating p53 degradation and reducing its protein stability. Coincidentally, TRIM28 can drive ubiquitination of p53 to suppress malignancy of tumors [25]. Furthermore, TRIM72 can bind directly to P53, facilitating its ubiquitination and subsequent degradation via the proteasome pathway, thereby decreasing trophoblast apoptosis [26]. The parallel roles of TRIM28 and TRIM72 in targeting p53 suggest that the TRIM family collectively contributes to p53 regulation in pregnancy disorders, opening new avenues for therapeutic targeting. Moreover, given that p53 activation enhances ROS production [27], TRIM28-mediated p53 suppression may indirectly limit ROS accumulation, providing an additional protective layer against placental oxidative stress.

However, several limitations should be noted. First, although the L-NAME–induced EOPE model mimics clinical features, it may not fully recapitulate the complexity of human EOPE. Second, the small number of animals used in this study may limit the statistical reliability of the results, and future studies with larger sample sizes are needed to validate the findings. In addition, the therapeutic potential of targeting TRIM28 requires further evaluation in clinical samples and larger animal models. Despite these limitations, our findings provide new insight into the molecular mechanisms of EOPE and highlight TRIM28 as a promising therapeutic target. Targeting TRIM28 may represent a novel strategy to preserve placental function and improve pregnancy outcomes in EOPE patients.

## 5 Conclusions

This study is the first to identify the critical role of TRIM28 in the pathogenesis of EOPE. We showed that TRIM28 expression is markedly reduced in EOPE placentas, which contributes to excessive trophoblast apoptosis and oxidative stress. Mechanistically, TRIM28 promotes p53 ubiquitination and degradation, thereby restraining p53-mediated pro-apoptotic signaling and ROS production. TRIM28 overexpression alleviated placental injury, preserved placental architecture, and improved hallmark clinical features of EOPE *in vivo*. These findings identify TRIM28 as both a regulator of trophoblast viability and a potential therapeutic target for EOPE. Targeting the TRIM28-p53 axis may represent a novel strategy to preserve placental function and improve pregnancy outcomes, offering a promising direction for future translational and clinical studies.
